# Robots in the clinical laboratory

**DOI:** 10.1155/S1463924688000033

**Published:** 1988

**Authors:** L. B. Roberts

**Affiliations:** Department of Pathological Biochemistry Gartnavel General Hospital 1053 Great Western Road Glasgow G12 OAH UK


Journal of Automatic Chemistry, Vol. 10, No. 1 (January–March 1988), pp. 4–5

### Robots in the clinical laboratory

**L. B. Roberts**

*Department of Pathological Biochemistry, Gartnavel General Hospital, 1053 Great Western Road, Glasgow G12 0AH, UK*

*The recent availability of robots specifically designed for use in laboratories raises the question 'Is there a place for robots in clinical chemistry laboratories?' Some direct experience with the Zymark robot over the last 18 months suggests that the answer to this question is equivocal and it is my intention to elaborate on this theme.*

Over the last 15 to 20 years there has been a steady increase in the use of automatic instruments in clinical chemistry laboratories, partly due to market exploitation by the instrument manufacturers and partly due to the greater clinical demand for diagnostic tests.

The clinical demand is such that a fairly large laboratory might be handling around 125 000 to 150 000 samples per year, representing about 2 million test results. The range of tests offered might be of 100 different types. Changes in workload arise from two sources; firstly from a general increase in established tests which is usually absorbed onto existing automatic equipment; and, secondly, from the addition of new tests to the laboratory repertoire. These are often complex in nature and, initially at least, are carried out in relatively small numbers. It is here where the use of robotic preparation units would have its greatest benefit and effect. Figure 1 shows the probable relationship between manual, robotic and dedicated instrumentation. As the complexity of a particular task increases, then the relative number of samples which can be handled by a human operator diminishes. Equally, as the complexity goes down, then the human operator can carry out more tasks of a repetitive nature. In the figure an arbitrary point of about 20 samples would define the point at which one could reasonably expect to carry out this analytical work on dedicated instrumentation. However, before instrument manufacturers will commission the manufacture of new automatic instruments they have to identify a market of sufficient size and consequently there is always an intermediate period during which the analytical laboratories find difficulty in meeting the clinical demands. It is here that the robot could have a major place.

It is worthwhile to examine the workload of a clinical laboratory and the operations necessary to complete that workload in order to identify areas of robotic application. The workload breakdown in the authors' laboratory is shown in table 1. Fully automated analyses are considered to be those which only require sample presentation to the equipment and in which a printed result is produced. Partially automated tests require preparative work ranging from 15 to 50% of the assay time and manual tests indicate significant preparative work before presentation of the specimen to an appropriate sensor

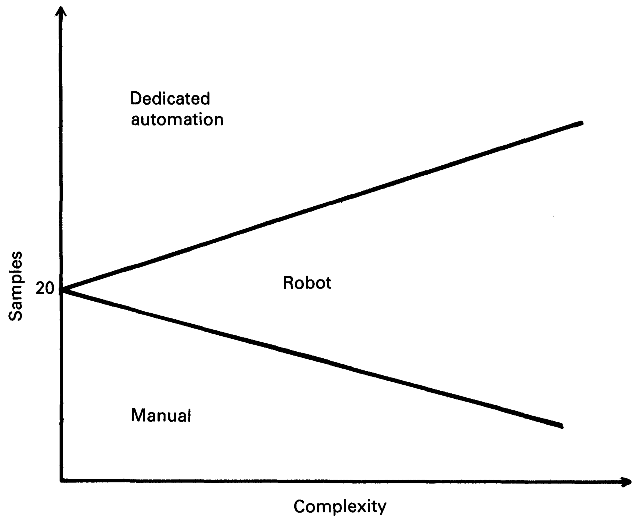

*Figure 1. Probable relationship between manual, robotic and dedicated instrumentation.*

instrument for quantitative measurements. In terms of the definitions applied, 90% of the laboratory workload is already fully automated, 3% partially automated and 7% manual. In a subset of the manual tests, 2% of the overall total are suitable for robotic application and these are indicated by an asterisk. It will be seen that all of these tests have a high preparative component and experience with the determination of free thyroxine using the Zymark robot suggests that maximum benefit would be obtained by using the robot for preparative work overnight handling up to 50 samples inclusive of calibrants and quality control. A detailed work study showed that when analysing the same number of specimens the operator time fell from 73 to 23 minutes when using the robot, which represents a useful saving. Conversely, the overall time for analysis rose from 160 min to 460 min, illustrating the relative slowness of the robotic procedure. Hence the use of the robot system overnight.

If one considers using the robot in this manner then an overall appraisal of the laboratory function should be undertaken. There is no real clinical reason why some of the common tests currently performed by the laboratory on a daily basis should not be delayed in order to facilitate better organizational programming of the analytical workload. This would mean that much of the preparative work associated with specific analyses would be carried out overnight. One should be able to clearly distinguish between most tests requiring a short turn round-time such as serum potassium, and the determination of vitamin A or alphafetoprotein which are not, under usual circumstances, required urgently and there is no clinical reason why these results could not be delayed for a period of 24 hours. This would effectively allow the preparative component of the analysis to be performed overnight

*Table 1. Workload.*

| | | |
|---|---|---|
| *Fully automated tests* | Sodium, potassium, chloride, bicarbonate, urea, creatinine, total protein, albumin, bilirubin, AST, ALT, alkaline phosphate, calcium, phosphate, iron, urate, drugs, GGT | 1968200 (89%) |
| *Partially automated tests* | Cholesterol, LDH, acid phosphate, C.K., IBC, specific proteins, triglycerides, urine (total protein, creatinine, urea), others | 74800 (3·4%) |
| *Manual tests* | Blood gases, electrophoresis, bilirubin*, glucose, thyroid function tests*, cortisol (B&U)* trace metals*, HbAlC*, tumour markers*, Lip.E.P., osmolality, oestrogen receptors*, urine (sodium, potassium, osmolality, nitrogen*, xylose*, NMA*, HIAA*), others | 156600 (7·1%) |
| | | *43200 (1·96%) |

without any significant loss in clinical usability. Our investigations showed that it is quite possible to program the robot to start working in the early hours of the morning and timed to provide a series of prepared samples for the estimation of free thyroxine or any other immunoassay which is then available to a member of staff for transfer to end sensor reading for quantitation by 09.00 hours. Of course the robot equipment is also available for use during the day and one of the potential applications is in the laboratory reception. Its use in this area is much more problematical because the sequence of preparative steps in handling blood samples contain so many value judgements that it would be difficult to see how a robot could be programmed to take care of such a wide variety of circumstances. Whilst a robotic system is quite capable of separating and centrifuging plasma or serum from red cells and distributing the resultant separated material into appropriate aliquots, the process of matching request forms with samples and making subjective assessments of the samples in so far as lipaemia, haemolysis or small volumes is concerned is not yet the province of a robot.

With a commitment by the laboratory to reorganize their working schedules then productivity should increase using a robot system. Given the present capital cost of appropriate robotic equipment, for example £30 000, it would be possible to absorb an additional workload equivalent to the revenue cost of one analyst (£7000) thereby given a notional payback period of approximately four to five years. With the present slow speed of operation it is unlikely that a single robot installation will be more than equivalent to one member of staff, even if the equipment is used on a two shift basis.

In order to facilitate robotic developments three levels of user can be identified. The first is that the programmer who must have a thorough understanding of how to program the system, *de novo*, and be able to string together the various subprograms which he or she develops. The second level is that of a supervisory member of staff who can take the existing subroutines and call them, via the robot controller, to produce a composite set of steps to provide a full analytical sequence. There will be considerable overlap between the functions of these two members of staff. The third level of user is that of the operator who would use a single code in order to call a specific program. Our experience would suggest it would be necessary to employ one or more programmers whose responsibilities should cover more than one hospital unit, with, in addition, sufficiently experienced supervisory staff to operate at the unit level.

I believe that the introduction of robots into clinical chemistry laboratories will occur slowly because since most of our analyses are performed on dedicated equipment the need to automate the residual tests is less imperative. However, the biggest hurdle will be the effort needed to reorganize the general working of the laboratory so that a significant proportion of the work can be performed overnight. People are reluctant to make changes in a system which works satisfactorily but in the present economic and political climate increases in productivity can be achieved by utilizing robot systems.

**PROCESS ANALYSIS**

A half-day meeting of the North East Region of the analytical Division of the Royal Society of Chemistry, will be held at 1.25 p.m. on Wednesday, 24 February 1988, in Lecture Theatre A, School of Chemistry, University of Hull, UK.

On-line process gas chromatography in a research and development environment
*J. Green (BP Chemicals Ltd, Hull).*

Process analysis in the steel industry
*W. R. Marris (British Steel, Scunthorpe).*

Sensors in process analysis applications
*H. Thompson (Kent Industrial Measurements Ltd, Stonehouse).*

Spectrophotometric field monitor for water quality parameters
*P. J. Worsfold and J. R. Clinch (University of Hull).*

*Details from Analytical Division, Royal Society of Chemistry, Burlington House, London W1V 0BN.*

